# Rat Spinal Cord Injury Associated with Spasticity Leads to Widespread Changes in the Regulation of Retained Introns

**DOI:** 10.1089/neur.2021.0042

**Published:** 2022-03-04

**Authors:** Samantha N. Hart, Samir P. Patel, Felicia M. Michael, Peter Stoilov, Chi Jing Leow, Alvaro G. Hernandez, Ariane Jolly, Pierre de la Grange, Alexander G. Rabchevsky, Stefan Stamm

**Affiliations:** ^1^ Department of Molecular and Cellular Biochemistry, University of Kentucky, Lexington, Kentucky, USA.; ^2^ Department of Physiology and Spinal Cord and Brain Injury Research Center (SCoBIRC), University of Kentucky, Lexington, Kentucky, USA.; ^3^ Department of Biochemistry, University West Virginia, Morgantown, West Virginia, USA.; ^4^ DNA Services Facility, University of Illinois, Urbana, Illinois, USA.; ^5^ GenoSplice Technology, Paris, France.

**Keywords:** gene expression, mRNA, pre-mRNA splicing, spasticity

## Abstract

To determine molecular changes that correlate with long-term physiological changes after spinal cord injury associated with spasticity, we used a complete transection model with an injury at sacral spinal level S2, wherein tail spasms develop in rats weeks to months post-injury. Using Illumina and nanopore sequencing, we found that from 12,266 expressed genes roughly 11% (1,342) change expression levels in the rats with spasticity. The transcription factor PU.1 (Spi-1 proto-oncogene) and several of its known regulated genes were upregulated during injury, possibly reflecting changes in cellular composition. In contrast to widespread changes in gene expression, only a few changes in alternative exon usage could be detected because of injury. There were more than 1,000 changes in retained intron usage, however. Unexpectedly, most of these retained introns have not been described yet but could be validated using direct RNA nanopore sequencing. In addition to changes from injury, our model allowed regional analysis of gene expression. Comparing the segments rostral and caudal to the injury site in naïve animals showed 525 differentially regulated genes and differential regional use of retained introns. We did not detect changes in the serotonin receptor 2C editing that were implicated previously in this spinal cord injury model. Our data suggest that regulation of intron retention of polyadenylated pre-mRNA is an important regulatory mechanism in the spinal cord under both physiological and pathophysiological conditions.

## Introduction

In addition to rendering paralysis, a common secondary complication of spinal cord injury (SCI) is the development of chronic abnormal spinal reflexes below the injury level that render uncontrolled muscle spasms.^1,2^ The underlying molecular changes correlated with long-term SCI are complex and remain uncertain.^3^ Recent models have implicated heightened constitutive serotonergic activities as well as general inflammation.^4–8^

Notably, most published ribonucleic acid sequencing (RNAseq) data in pre-clinical SCI models have been derived from acute and subacute time points, before the development of fulminant spasticity.^9–12^ Currently, data on SCI-induced chronic changes in gene expression leading to spasticity are lacking. To gain further insight into the molecular mechanisms, we used the chronic sacral (S2) spinal cord transection model in rats that has quantifiable tail spasticity develop in response to tactile or nociceptive stimuli like stretch, pinch, or electric shock.^1,13,14^

Because a transection model clearly defines the site of injury, we analyzed the changes in gene expression in tissues that were rostral and caudal to the S2 injury at a time when spasms were well established. We next used Illumina and Oxford Nanopore sequencing to compare injured and naïve segments above and below the S2 level, extending previous molecular characterization studies^5,11^ that assessed tissue at or below the injury site. Our analysis revealed changes in transcription, which in part reflected a change in cellular composition.

A subset of these programs emanates from the PU.1 (Spi-1 proto-oncogene) transcription factor that has been found deregulated in a mouse contusion SCI model^11^ and that is upregulated in activated microglia,^15^ indicating commonalities among different injury models. Surprisingly, we observed almost no changes in alternative exon usage. Supported by nanopore sequencing, however, which analyzes whole polyadenylated mRNA molecules, we unexpectedly detected thousands of alternatively retained introns that show region- and injury-specific differences in expression, which so far have not been described. Thus, retaining introns in polyadenylated RNA emerges as a regulatory mechanism in the spinal cord, contributing to region-specific changes and changes from injury.

## Methods

### SCI model

All animal housing conditions, surgical procedures, and post-operative care were conducted according to the University of Kentucky Institutional Animal Care and Use Committee and the National Institutes of Health animal care guidelines (animal protocol #733M2004). Adult male Sprague-Dawley rats were anesthetized with a combination of ketamine (80 mg/kg, Zoetis, Parsippany-Troy Hills, NJ) and xylazine (10 mg/kg, Akorn, IL), and under sterile conditions the spinal cord was exposed at the S2 spinal level after upper lumbar laminectomy. After topical administration of 2% lidocaine, (Henry Schein, Cleveland, OH), micro scissors were used to carefully incise the dura and transect the spinal cord at the S2 level. Care was also taken to prevent damage to the anterior artery or posterior vein to prevent ischemia of the spinal cord below the incision, which leads to flaccid tail paralysis.

To ensure complete S2 transection, resected residual tissue was gently aspirated with glass micropipettes before the dura was sealed with a thin 0.5% agarose sheet. Wounds were then irrigated with sterile saline, the muscle layers sutured using 3-0 Vicryl (Ethicon, San Lorenzo, Puerto Rico), and skin openings stapled with Michel wound clips (Fine Science Tools, Foster City, CA). Injured rats were housed one per cage with food and water *ad libitum,* injected subcutaneously (sc) with 5 mL lactated Ringer solution for fluid replacement, and placed on a heating pad during recovery. Injured rats received analgesic buprenorphine (0.03 mg/kg, sc, Reckitt Benckiser Healthcare, Hull, England) twice a day for three days and antibiotic (33.3 mg/kg Cefazolin, sc, WG Critical Care, NJ) twice daily for five days, along with 5 mL Ringer lactate.

A 5-point ordinal scale was used to measure tail spasm severity based on the degree of curling, development of clonus, and hyperreflexia.^1–3^ The injured animals (*n* = 7) developed fulminant muscular spasticity of the paralyzed tail within 30 days; those with a flaccid tail (*n* = 1) were excluded from all analyses. To confirm and quantify the development of spasticity, injured animals underwent electromyography (EMG) analysis following a 0.1 mV electrical stimulation of the tail after 60 days post-injury.

At the terminal time points (∼24 weeks post-injury), animals were euthanized by CO_2_ inhalation followed by decapitation. The injured spinal cords were quickly re-exposed and the spinal cord segments below (∼7 mm) and above (∼5 mm) the S2 transection site were dissected, immediately frozen on dry ice, and stored at -80°C until RNA isolation. For naïve samples (*n* = 7), equivalent segments of uninjured spinal cord were similarly processed.

### RNA isolation

Total RNA was isolated from the sections of rat spinal cord using the RNeasy Mini Kit (Qiagen, Hilden, Germany). Each sample was weighed and kept frozen on dry ice before total RNA isolation. Spinal cord samples were homogenized in Qiazol (Qiagen) using 5 mL Dounce homogenizers. Instead of eluting the RNA once as per the manufacturer's protocol, the total RNA was eluted three times in 30 μL water in separate tubes and the elutions combined once the concentrations were determined by Qubit (Invitrogen, Needham, MA). Total polyA+ RNA was selected for nanopore sequencing using Dynabeads™ mRNA Purification Kit (ThermoFisher, Needham, MA).

### Construction of strand specific RNAseq libraries for Illumina sequencing

Total RNAs were run on a Fragment Analyzer (Agilent, Santa Clara, CA) to evaluate RNA integrity. The RNAseq libraries were constructed with the TruSeq Stranded mRNA Sample Prep kit (Illumina, San Diego, CA). Briefly, polyadenylated messenger RNAs (mRNAs) were enriched from 500 ng of high-quality deoxyribonucleic acid (DNA)-free total RNA with oligodT beads. The mRNAs were chemically fragmented, annealed with a random hexamer, and converted to double stranded cDNAs, which were subsequently blunt-ended, 3'-end A-tailed, and ligated to indexed adaptors.

Each library was ligated to a uniquely dual indexed adaptor (unique dual indexes) to prevent index switching. The adaptor-ligated double-stranded cDNAs were amplified by polymerase chain reaction (PCR) for eight cycles with the Kapa HiFi polymerase (Roche, CA) to reduce the likeliness of multiple identical reads because of preferential amplification. The final libraries were quantified with Qubit (ThermoFisher), and the average library fragment length was determined on a Fragment Analyzer. The libraries were diluted to 10 nM and further quantitated by qPCR on a CFX Connect Real-Time qPCR system (Biorad, Hercules, CA) for accurate pooling of the barcoded libraries and maximization of number of clusters in the flow cell.

### Sequencing of libraries in the NovaSeq

The libraries were sequenced from both ends of the fragments for a total of 150 nt from each end in a NovaSeq SP flow-cell (Illumina). The fastq read files were generated and demultiplexed with the bcl2fastq v2.20 Conversion Software (Illumina). The quality of the demultiplexed fastq files was evaluated with the FastQC software, which generates reports with the quality scores, base composition, k-mer, GC and N contents, sequence duplication levels, and overrepresented sequences.

### Illumina data analysis

The RNAseq data analysis was performed by GenoSplice technology (GenoSplice, Paris, France). Analysis of sequencing data quality, reads repartition (e.g., for potential ribosomal contamination), inner distance size estimation, genebody coverage, strand-specificity of library were performed using FastQC, Picard-Tools, Samtools, and RSeQC. Reads were mapped using STAR^16^ on the rn6 genome assembly.

Gene expression was estimated as described previously^16–19^ using Rat FAST DB v2018_1 annotations. Only genes expressed in at least one of the two compared conditions were analyzed further. Genes were considered as expressed if their fpkm (fragments per kilobase of transcript per million mapped reads) value was greater than fpkm of 95% of the intergenic regions (background). Analysis at the gene level was performed using Deseq2.^20^ Genes were considered differentially expressed for fold-changes ≥1.5 and uncorrected *p* values ≤0.05.

Pathway analyses and transcription factor network analysis were performed using WebGestalt^21^ merging results from upregulated and downregulated genes only, as well as all regulated genes. Pathways and networks were considered significant with *p* values ≤0.05.

A deconvolution analysis was performed using GEDIT^22^ using a dedicated signature.^23,24^

Analysis at the splicing level was first performed taking into account only exon (or intron) reads (“EXON” analysis) to potentially detect new alternative events that could be differentially regulated (i.e., without taking into account known alternative events). Analysis at the splicing level was also performed by taking into account known patterns (“PATTERN” analysis) using the FAST DB splicing patterns annotation (i.e., for each gene, all possible splicing patterns were defined by comparing exon content of transcripts).

All types of alternative events were analyzed: alternative first exons, alternative terminal exons, cassette exons, mutually exclusive exons, alternative 5' donor splice site, alternative 3' acceptor splice sites, intron retention, internal exon deletion, and complex events corresponding to mix of several alternative event categories). EXON and PATTERN analyses were based on the splicing-index calculation as described previously.^17,19^ Results were considered statistically significant for *p* values ≤0.05 and fold-changes ≥1.5 for PATTERN analysis and *p* values ≤0.01 and fold-changes ≥2.0 for EXON analysis. Finally, significant results from EXON and PATTERN analyses were merged to obtain a single results list.

Another splicing analysis dedicated for retained introns was performed as described previously.^25^ Donor and acceptor splice site scores were calculated using MaxEntScan.^26^ Enriched sequence motif analysis was performed using DREME.^26^ The editing status of 5HT2c transcripts was performed using Samtools mpileup.

### Nanopore sequencing

Before preparing the polyA RNA for nanopore sequencing, the concentration of the sample was determined by Qubit (ThermoFisher). The cDNA for sequencing was prepared using the Direct RNA Sequencing Kit (Oxford Nanopore Technologies, Oxford, UK) using FLO-MIN106D flow cells. The MinION cells were run for 72 h at 180 volts for each sample, and a new flow cell was used for each sample. Basecalling was performed after the runs were completed.

### Nanopore data analysis for retained intron validation

Basecalling was performed using Guppy (Oxford Nanopore Technologies). Read correction using Illumina samples was performed using TALC. Quality control of data was performed using FastQC, Samtools, RSeQC, and NanoPlot.^27^ Reads were aligned using Minimap2.^28^ The validation of retained introns was performed using custom Perl scripts.

## Results

### RNA isolation from an S2 spinal cord transection model after development of spasticity

**FIG. 1.**
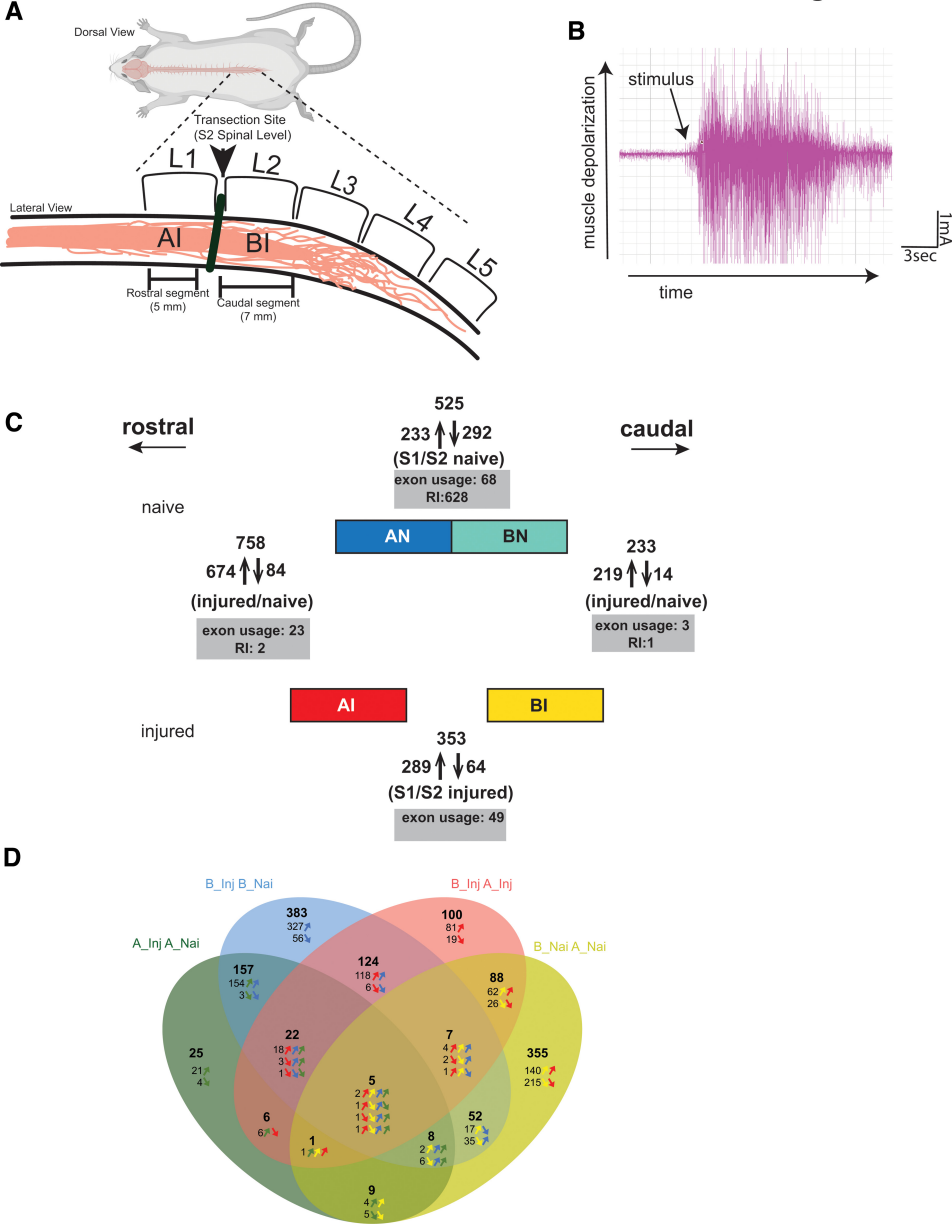
Rat spinal cord transection animal model leading to spasticity. (**A**) Animal model: a transection was performed between the sacral spinal cord segments S1 and S2 underneath the lumbar vertebrae L1 and L2, which leads to tail spasms after sixty days. At ∼180 days post-injury, 7 mm of spinal cord corresponding to the caudal region below the injury (BI) and 5 mm of spinal cord corresponding to the rostral region above the injury (AI) were collected. An assessment of the spasticity is shown in Supplementary Figure 1. Non-injured animals were dissected similarly, and the tissues are denoted as AN, above naïve, and BN, below naïve. (**B**) Representative electromyogram (EMG) showing rat tail spasticity induced by electrical stimulation. Arrow indicates the time point of stimulus. (**C**) Overview of the changes in gene expression. Four conditions were compared: (1) injured rostral segment with injured caudal segment; (2) injured caudal segment with naïve caudal segment; (3) naïve rostral segment with naïve caudal segment, and (4) injured rostral segment with naïve rostral segment. Numbers indicate the changes in gene expression, and arrows indicate whether upregulation or downregulation. Shaded boxes indicate the changes in exon usage. RI: retained introns. (**D**) Comparison of changes among the four conditions described in panel C. The Venn diagram shows the number of gene expression changes common to each condition, indicated by coloring overlaps. Arrows indicate the direction of the changes. A_Inj A_Nai: A injured compared with A naïve; B_Inj B_Nai: B injured compared with B naïve; B_Inj A_Inj: B injured compared with A injured; B_Nai A_Nai: B naïve compared with A Naïve.

To determine changes in gene expression after SCI, we used an S2 transection model. After laminectomy of lumbar vertebrae L2, a resection was made between sacral spinal levels S1 and S2, located between lumbar vertebra L1 and L2. In contrast to rat contusion models used to generate gene expression data at the injury epicenter,^29^ this S2 transection model allowed the specific analysis of regions above and below the injury (Fig. 1A). Animals were kept for up to 185 days, by which time tail spasticity developed fully, determined by tail pinch and EMG (Supplementary Fig. S1, Fig. 1B).

Spinal cord tissue from 0.7 cm below (caudal to) the injury (caudal segment, below injury [BI]) and 0.5 cm above (rostral to) the injury (rostral segment, above injury [AI]) was dissected and nerve endings removed.

The RNA from at least three naïve and three injured rats was analyzed using Illumina sequencing employing 150 nt long base-paired reads. All samples showed good quality, (Supplementary Fig. S2A–C). On average, the samples gave 62,843,730 reads, from which 88.06% could be uniquely mapped to the rat genome. Of the reads, 98% were on the expected strand, and on average each sample expressed 12,266 genes. Seven genes represented more than 0.5% of the reads. The highest expressed gene was myelin basic protein (MBP, 1.9% of all reads), followed by the mitochondrial nicotinamide adenine dinucleotide (NADH) dehydrogenases Nd1, Nd4, Nd2, the stearoyl-CoA desaturase Scd2, the matrix associated protein SPARCL1, and apolipoprotein E (ApoE).

### Chronic injury causes predominant changes in gene expression and only a few changes in alternative exon usage

We next compared differences in gene expression in the four conditions: tissue corresponding to rostral and caudal segments—i.e., above and below the injury, both naïve and injured. We refer to these tissues as AI, AN, BI, and BN (above injury, above naïve, below injury, below naïve). Each condition was represented by at least three samples from independent animals. Using a fold change of larger than 1.5 and a *p* value less than 0.05 as the cutoff criterion as in previous studies,^16–19^ we found 1,342 changes in gene expression (Fig. 1C,D) when analyzing all four conditions.

A comparison of the injured with naïve caudal segments showed 219 genes upregulated and 14 genes downregulated, the largest change being a 4.16 upregulation of Lgals3, the carbohydrate binding protein Galectin 3. Pathway and gene ontology analysis showed that numerous deregulated genes functioned in systems related to the immune system. The transcription factor network analysis showed an enrichment of genes regulated by the transcription factors erythroblast transformation specific 2 (ETS2), ELF1, and PU.1. Interestingly, PU.1 (Spi-1 proto-oncogene) itself is deregulated during injury, suggesting it could be master-regulator of the injury response (Supplementary Table S1). PU1 is implicated in myeloid maturation especially differentiation and activation of macrophages and cytokine regulation^30,31^ and is increased three days after SCI in microglia.^15^

A similar analysis comparing the changes between injured and naïve spinal cords above the injury site in the rostral segment showed 674 upregulated and 84 downregulated genes. The matrix metalloproteinase Mmp12 was most deregulated as it was exclusively expressed in injured samples. Mmp12 breaks down the extracellular matrix, especially elastin, during tissue remodeling. The pathway analysis indicated an enrichment of genes acting in osteoclast differentiation, immune response, and steroid biosynthesis. Similar to BI changes, transcription factor analysis showed a strong enrichment of genes targeted by PU.1, corresponding to the upregulation of the PU.1 transcription factor during injury (Supplementary Table S2).

There were only a few changes in alternative exon usage that included three changes in cassette exons and one change in intron retention below the injury. Above the injury, a total of 25 exons were changed that included two retained introns (Table 1).

**Table 1.** Summary of Changes in Gene Expression under Various Conditions

A. Regulated Genes caudal injured vs. caudal naïve (BI vs. BN)			
	Upregulated	Downregulated	All
Number of regulated genes	219	14	233
Max fold change	4.16	1.79	4.16
Gene with the max fold change	Lgals3	*X95721*	Lgals3

Alternative event type	Number of regulated events		
Alter. First Exon	0		
Alter. Terminal Exon	1		
Exon Cassette	1		
Mutually Exclusive Exons	0		
Alter. Acceptor Splice Site	0		
Alter. Donor Splice Site	0		
Intron Retention	0		
Internal Exon Deletion	1		
Complex	0		
Unknown (# introns)	1 (1)		

B. Regulated genes rostral injured vs. rostral naïve (AI vs. AN)			
	Upregulated	Downregulated	All
Number of regulated genes	674	84	758
Max fold change	517.74	27.53	517.74
Gene with the max fold change	Mmp12	RT1-CE16	Mmp12

Alternative event type	Number of regulated events		
Alter. First Exon	8		
Alter. Terminal Exon	2		
Exon Cassette	2		
Mutually Exclusive Exons	1		
Alter. Acceptor Splice Site	0		
Alter. Donor Splice Site	1		
Intron Retention	1		
Internal Exon Deletion	0		
Complex	0		
Unknown (# introns)	10 (1)		

C. Regulated genes rostral injured vs. caudal injured (AI vs. BI)			
	Upregulated	Downregulated	All
Number of regulated genes	289	64	353
Max fold change	14.38	6.6	14.38
Gene with the max fold change	*DQ615084*	RT1-Ba	*DQ615084*

Alternative event type	Number of regulated events		
*Alter. First Exon*	*22*		
Alter. Terminal Exon	7		
Exon Cassette	2		
Mutually Exclusive Exons	0		
Alter. Acceptor Splice Site	0		
Alter. Donor Splice Site	0		
Intron Retention	1		
Internal Exon Deletion	0		
Complex	0		
Unknown (# introns)	29 (11)		

D. Regulated genes rostral naïve vs. caudal naïve (AN vs. BN)			
	Upregulated	Downregulated	All
Number of regulated genes	233	292	525
Max fold change	4.86	6.04	6.04
Gene with the max fold change	Reg3b	*DQ608544*	*DQ608544*

Alternative event type	Number of regulated events		
Alter. First Exon	26		
Alter. Terminal Exon	19		
Exon Cassette	1		
Mutually Exclusive Exons	0		
Alter. Acceptor Splice Site	0		
Alter. Donor Splice Site	0		
Intron Retention	17		
Internal Exon Deletion	0		
Complex	0		
Unknown (# introns)	632 (611)		

AI, above injury; BI, below injury; AN, above naïve; BN, below naïve.

Functionally, genes expressed differentially between naïve and injured regions frequently correspond to changes in B and T cell activation, along with inflammation pathways (Supplementary Tables S1–S4).

### Tissue above and below the injury reacts differently to injury

Because we used a spinal cord transection model, we could compare the response in tissue located above and below the injury site. When injured and naïve samples are compared, there are 758 AI changes and 233 BI changes. When comparing injured rostral with injured caudal segments, 289 genes were upregulated and 64 genes were downregulated. The strongest upregulated gene was rat-specific. From genes with human homologs, the thyroid hormone transporter Ttr (transthyretin) showed a 12.7-fold upregulation in rostral samples while RT-Ba (rat ortholog to the major histocompatibility complex) showed the largest 6.6-fold downregulation (Supplementary Table S3). Thus, depending on location relative to the injury, the spinal cord exhibits different changes in gene regulation.

### Changes in gene expression after injury correlate with a change in cellular content

Because our model determined the chronic effects of an injury ∼24 weeks after the transection, it is possible that the changes in gene expression reflect remodeling of tissue and thus a change in cellular composition. We performed deconvolution analyses with GEDIT (Gene Expression Deconvolution Interactive Tool)^22^ using neuronal signatures and investigated single cell signatures from brain vasculature.^23,24^

**FIG. 2.**
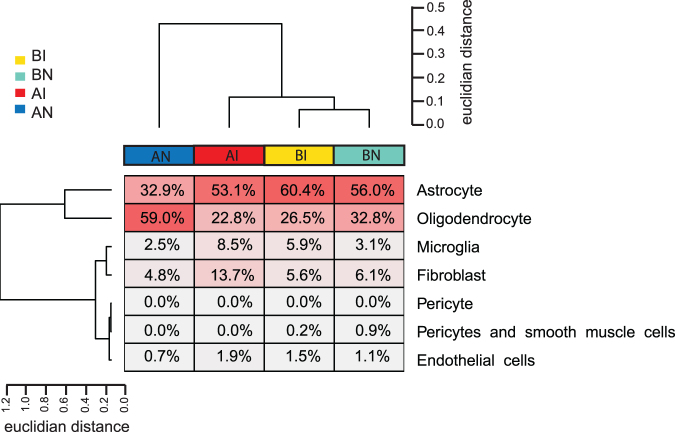
Cluster analysis of overall gene expression. The unsupervised clustering of expressed genes was compared with the gene expression signatures of cell types indicated. Euclidian distance is the square root of the sum of the squared differences in gene expression. BI, below injury; BN, below naïve; AI, above injury; AN, above naïve.

All samples showed RNA expression from mostly ventral inhibitory VI-1 and dorsal Inhibitory DI-5 and DI-4 neurons. Overall, injury changed cellular content from astrocytes to oligodendrocytes in rostral segments (mean of 59.0 ± 26.2% of oligodendrocytes and 32.9 ± 18.9% of astrocytes in rostral naïve vs. 22.8 ± 4.9% of oligodendrocytes and 53.1 ± 6.1% of astrocytes in rostral injured) and also increased either the number or expression from fibroblasts (13.7 ± 8.3% vs. 4.8 ± 4.0%), microglia (8.5 ± 2.7% vs. 2.5 ± 2.4%), and endothelial cells (1.9 ± 0.4% vs. 0.7 ± 1.1%), (Fig. 2). Similar changes occurred in the caudal segments but were less pronounced. Thus, in agreement with the known inflammatory response to SCI,^32^ changes resulting from injury reported here reflect, in part, different cellular compositions.

### Gene expression differs above and below S2 in naïve sacral spinal cord

Given the differences between the rostral and caudal segments in response to injury, we next asked what the differences between these segments in naïve animals were. There were 525 differences in gene expression, with 233 genes stronger and 292 genes weaker expressed in the rostral segments. Surprisingly, there were more differences in expression between naïve spinal levels than between injured and naïve caudal S2 segments. Most of these changes were region-specific and corresponded to multiple functions, defined by gene ontologies. The greatest difference was seen for a member of the lectin superfamily, regenerating islet-derived 3 beta (Reg3A) that was 4.9-fold higher expressed in rostral S1 segments. The noncoding gene MIR338 showed a 5.1-fold weaker expression in caudal S2 segments. The MIR338 hosts a miRNA that regulates cytochrome c oxidase (COX) subunit 4. Thus, the spinal cord shows region-specific gene expression.

### The number of retained introns differs above and below S2 in naïve sacral spinal cord

Unexpectedly, a large difference in the dataset was the usage of retained introns between experimental groups. Differentially regulated intron retention has been observed during meiosis in germ cell differentiation, where meiotic spermatocytes generate a large set of genes with retained introns that stay in the nucleus for several days, until the introns are removed post-meiosis.^17^ Similarly, neurons retain introns in nuclear poly(A) RNA that are rapidly removed after neuronal activation, leading to their transport into the cytosol and translation. This mechanism allows for a rapid release of mRNAs made from large genes, as only a single retained intron needs to be removed.^25^

When comparing naïve rostral and naïve caudal segments, 628 retained introns in 462 genes showed changes in expression (Supplementary Table S4). Most of these introns (622) showed higher expression levels in caudal segments. With a fold-change decrease of 12.5, intron 7 of adenosine triphosphate (ATP) binding cassette subfamily A member 2 (Abca2) showed the largest difference. An intron in the KH-domain containing gene QK1 (quaking) showed a 3.5-fold upregulation in the rostral segment. QK1 binds to RNA and has a role in myelination, regulating myelin associated glycoprotein and myelin basic protein processing,^33^ which could thus reflect different cellular composition. This appears to correlate with the observed phenotypic switch from astrocytes to oligodendrocytes above the injury, perhaps signifying remyelination of new neural circuitry. Overall, these genes with intron retention-mediated regulation were involved in metabolic pathways, especially fatty acid metabolism and transport of small molecules (Supplementary Table S4).

When comparing rostral and caudal segments after injury, we could detect changes in only 12 retained introns (Supplementary Table S3), suggesting that a differential usage of retained introns is a hallmark of chronic SCI.

The regulated retained introns did not reside in genes that were differentially expressed between rostral and caudal segments—i.e., underwent a promoter regulation. They thus represent an independent, novel mode of regulation that is in naïve animals more prevalent than the regulation by gene expression. The retained introns do not support the open reading frame of the mRNA when inserted into mRNAs and, thus, generate either truncated protein variants or destine the mRNA to nonsense mediated decay.^25^

In addition to more introns being retained in the naïve caudal compared with naive rostral segments, retained introns were seen throughout all samples, and >98% were located in sense orientation, which together rules out DNA contamination.

### Regulatory features of retained introns

**FIG. 3.**
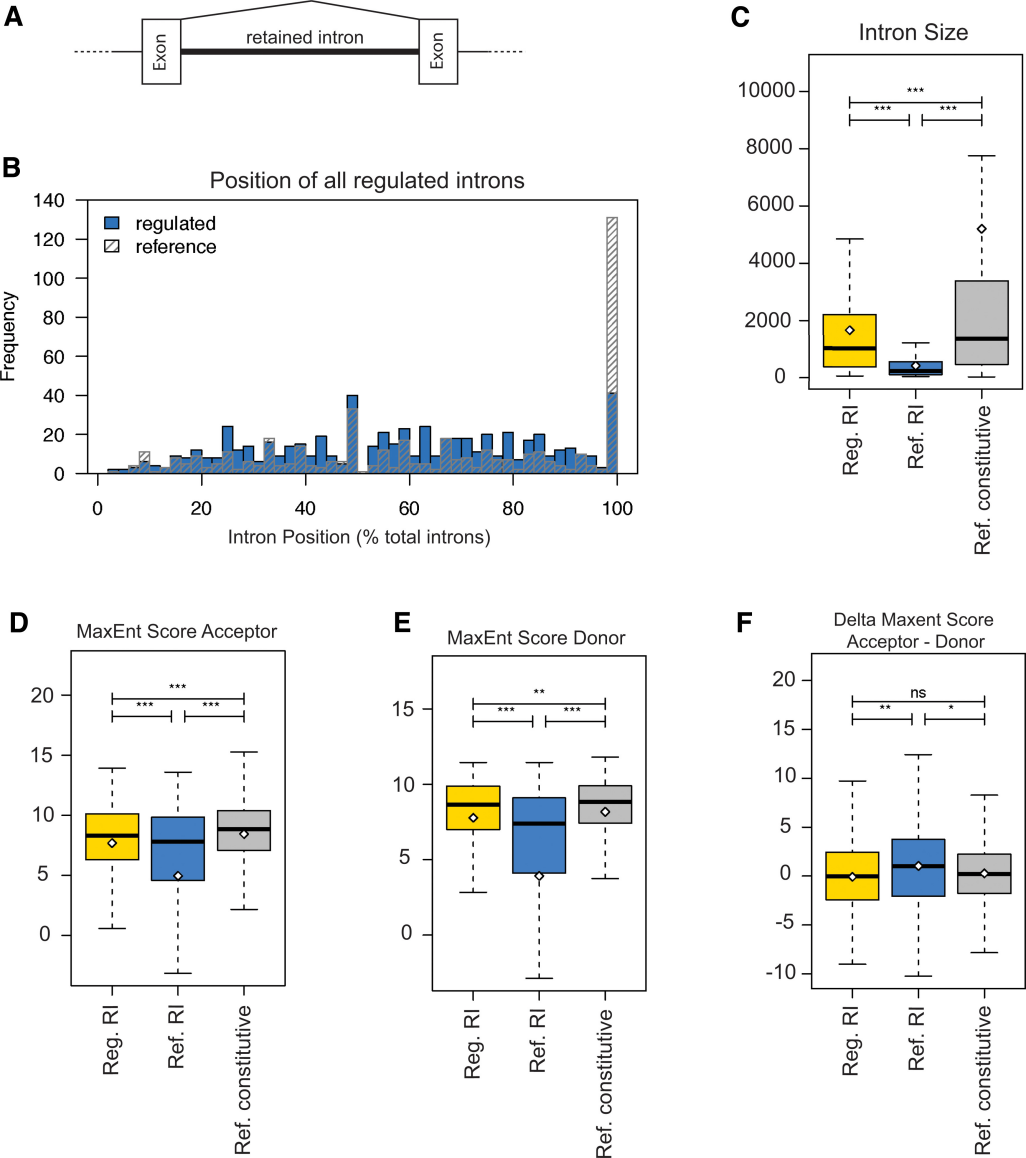
Properties of retained introns (RI) regulated during spinal cord injury. (**A**) Structure of a RI (thick line) that can be either included (thick line) or spliced out (thin lines). (**B**) Position of regulated RIs in spinal cord. The position is indicated as percent of the total length of the pre-messenger ribonucleic acid. Reference is the position of rat RIs that do not undergo spinal cord regulation. (**C**) Length of the RIs. Reg RI: RIs differently regulated between rostral and caudal segments. Ref RI: RIs from the FAST DB database^54, 55^; Ref const introns: constitutively spliced introns. (**D–F**) Splice site strength of regulated RIs expressed as maximum entropy score for the acceptor (3' splice site) and donor (5' splice site). Reg. RI: RIs regulated in spinal cord; Ref RI: all known rat RIs; Ref- constitutive: all constitutive introns.

We next analyzed possible regulatory features of the regulated retained introns. For comparison, we determined the location of 459 known retained rat introns in genes expressed in the caudal or rostral samples that are not differentially included and found that they cluster at the 3' end of pre-mRNAs. In contrast, retained introns regulated in the spinal cord are predominantly located inside genes, peaking around 60% of the pre-mRNA length (Fig. 3A,B). Thus, overall, these retained introns would truncate the proteins in the middle if translated or destine the mRNA to nonsense mediated decay if exported from the cytosol.

Regarding possible regulatory features, we first determined retained intron length and found that the regulated introns are significantly longer than known unregulated retained introns, but shorter than constitutively removed introns (Fig. 3C). In addition, they are flanked by shorter exons, likely reflecting their internal location.

Their splice sites, especially the donor 5' splice site, adhere stronger to the mammalian consensus than other retained introns (Fig. 3D–F). There is no difference, however, between these introns and known retained or constitutive introns regarding branch point composition or poly pyrimidine tract features.

To identify possible common regulatory features, we compared regulated retained introns with all known rat retained introns and constitutively spliced introns (Supplementary Fig. S3A). We identified five putative RNA binding motifs that were located within the intron and enriched for introns expressed above the injury when compared with known retained or constitutive introns. Among the top motifs was ATAGA that resembles a suboptimal polyadenylation signal (consensus AATAAA). This motif is present in most regulated introns, both injured and naïve (Supplementary Fig. S3B,C). The motif is underrepresented in non-regulated retained introns. It is distributed non-evenly throughout the introns, but clusters at the 3' end of the intron (Supplementary Fig. S3D). Thus, regulated retained introns have characteristic sequence motifs that distinguish them from constitutive and non-regulated retained introns.

### Identification of a large number of previously unknown retained introns in rat spinal cord

**FIG. 4.**
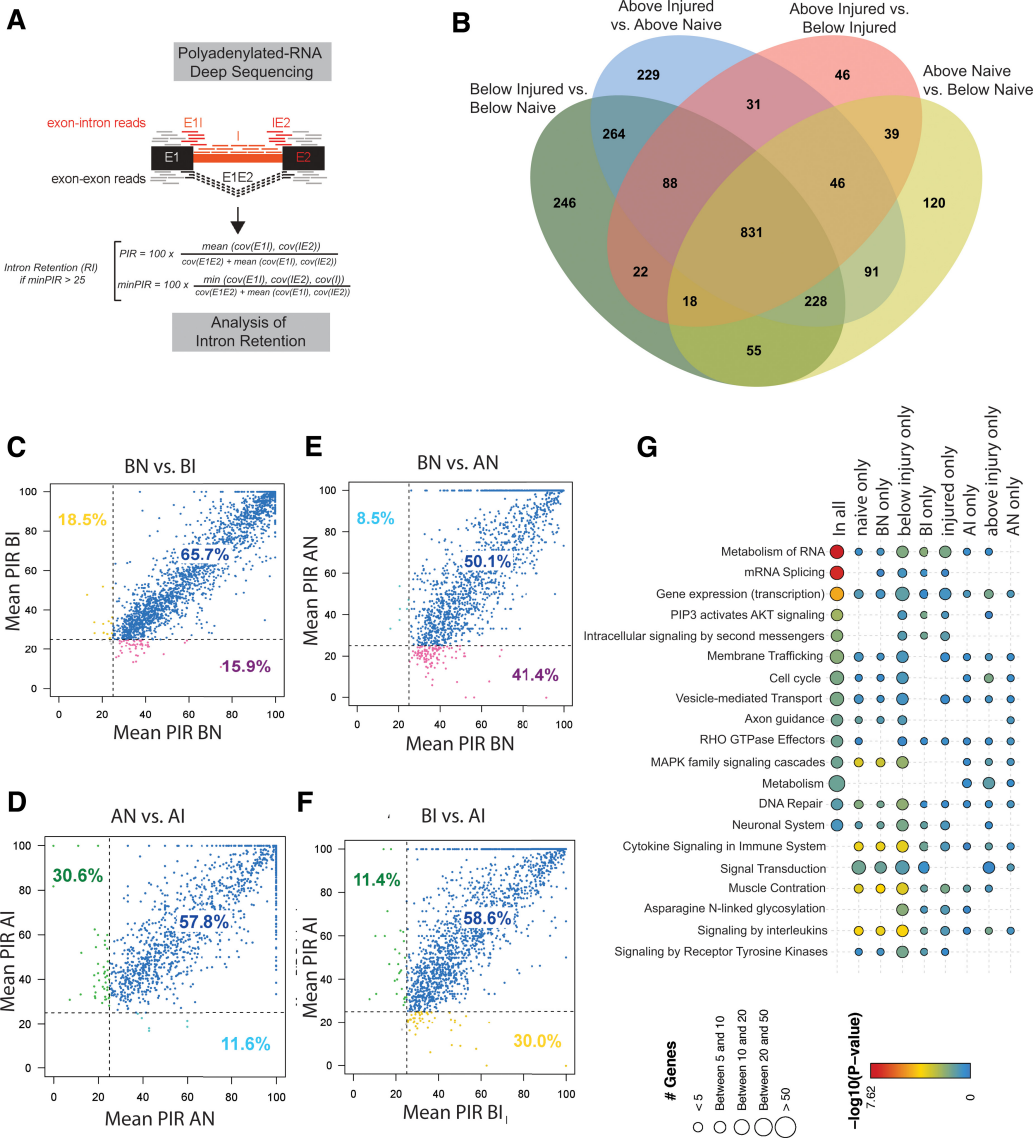
Identification of novel retained introns in rat spinal cord. (**A**) Strategy to identify novel retained introns in the dataset. (**B**) Venn diagram showing the expression of novel retained introns and their overlap with experimental conditions in the dataset. (**C–F**) Comparison of the percent intron retention (PIR) of the various experimental conditions. The PIR was calculated according to panel A. (**G**) Association of novel regulated retained introns with metabolic pathways. The number of regulated genes is indicated by the size of the circle and the regulation through the coloring, as shown in the legend below. The color scheme shows the p value of changes in the metabolic pathways. A lower p value with a warmer color indicates a higher likelihood for significance of the annotated change. BI, below injury; BN, below naïve; AI, above injury; AN, above naïve.

Because our analysis uncovered differentially regulated retained introns, we asked whether the sample set contained other, previously unknown retained introns. We reanalyzed the RNAseq data using a previously developed algorithm^25^ (Fig. 4A). Briefly, we calculated intron retention as the ratio (PIR, percent intron retention) between exon-intron-junction reads to the combined number of exon-junction and intron-junction reads.

For quantification, the minPIR ratio of exon-intron junctions and intronic reads to exon junction and intron junction reads was calculated (Fig. 4A). Introns were considered as retained with minPIR >25, with the intron expressed in more than 50% of the biological replicas, and with the hosting gene expressed. Using these conditions, we identified 2,354 novel retained introns in our dataset (Fig. 4B, Supplementary Table S5). About 35% (831) of these introns are common to all experimental conditions. Most introns specific for an experimental condition were detected in the naïve caudal sacral segments.

We next compared the percent inclusion of the novel retained introns across all four experimental sample groups. For the majority of introns (>50%), there was no change in regulation. Comparisons across experimental condition pairs, however, showed that up to 41% of retained introns exhibit a different inclusion ratio defined as intron usage of lesser or equal than 25% in one condition and more than 25% in the other condition.

Again unexpectedly, the most pronounced difference was between the naïve rostral and caudal segments (Fig. 4E). In addition, there were pronounced changes between the injured segments (Fig. 4C,D–F).

### Nanopore sequencing shows that most retained introns reside in polyadenylated RNA

A comparison with public databases (FAST DB [V2018_1]) showed that more than 90% of the introns identified by our algorithm (Fig. 4A) have not been described previously. To further validate their existence, we performed nanopore sequencing. Because direct RNA nanopore sequencing requires at least 1 μg of polyadenylated RNA, we pooled all previously analyzed RNAs from each experimental group, isolated polyA(+) RNA, and performed nanopore sequencing.

In nanopore sequencing, polyadenylated RNA is directly sequenced, and the sequencing tracks of a single molecule are queried. Because entire molecules are sequenced, the connectivity of exons and introns is known. We could validate up to 44% of introns predicted from Illumina sequencing. The lowest validation rates were in the samples from injured animals, possibly because of deadenylation of the transcripts from injury. Of note is that nanopore sequencing uses polyadenylated RNA. Thus, a large proportion of the novel identified retained introns reside in polyadenylated RNAs.

A functional characterization of the retained introns showed that all of them had stop codons in at least one reading frame, and more than 85% of the introns have stop codons in all three reading frames. A pathway analysis showed that the majority of the genes showing retained introns act in RNA metabolism, mRNA splicing, and transcription (Fig. 4G). The number of retained introns for genes involved in RNA metabolism and RNA splicing increases after injury both in rostral and caudal segments.

In summary, our data indicate that the spinal cord harbors polyadenylated RNAs containing thus far unknown retained introns. These introns will prevent translation and possible nuclear export of the RNAs, and their differential usage represents a novel response of pre-mRNA processing regulation to SCI.

### S2 transection does not result in changes of the serotonin receptor 2C (5HT2C) pre-mRNA editing or splicing

It has been reported previously that SCI changes pre-mRNA editing of the serotonin receptor 2C (5HT2C) and might contribute to chronic spasticity after the S2 injury.^4,34–39^ In addition, other serotonergic receptors have been implicated.^7,40^ The 5HT2C pre-mRNA generates two splicing isoforms via alternative splicing of exon Vb. In addition, the 5HT2C pre-mRNA undergoes RNA adenosine to inosine editing at five sites A–E.

**FIG. 5.**
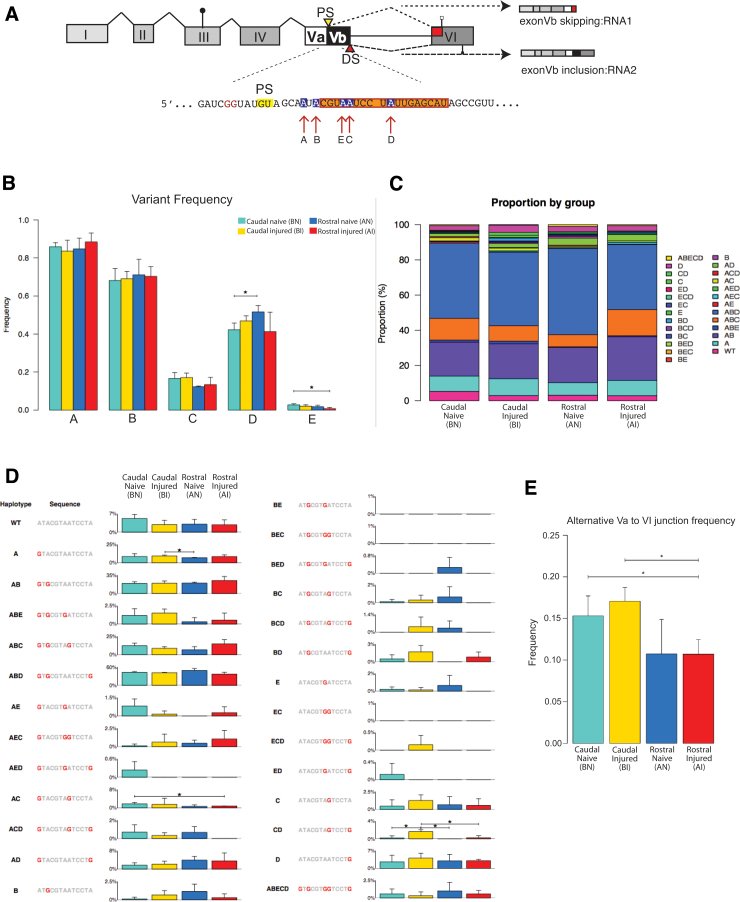
Pre-messenger ribonucleic acid (pre-mRNA) processing of the serotonin receptor 2C (5HT2C). (**A**) Schematic overview of the 5HT2C receptor pre-mRNA. Exons are indicated by roman numbers. Exon Vb is alternatively used, depending on recognition of the distal (DS) or proximal (PS) splice site. Skipping of exon Vb leads to RNA1 encoding a truncated receptor, and its inclusion generates RNA2 that includes the full-length receptor. Exon Vb contains five editing sites (A–E) that can be deaminated from adenosine to inosine. (**B**) Frequency of RNA editing at given sites under the four experimental conditions, as illustrated in Figure 1C. **(C**) Distribution of editing variants at different experimental conditions. The sum of all variants was set to 100%. (**D**) Distribution of the combination of editing sites. The sequences are indicated on the left, the frequency is shown on the right, as percent of all 5HT2C reads containing the editing sites. (**E)** Frequency of exon Vb skipping under the four experimental conditions. BI, below injury; BN, below naïve; AI, above injury; AN, above naïve.

The combination of RNA editing and alternative splicing generates at least 64 mRNA isoforms. Because an inosine is translated as a guanosine, A->I editing changes the encoded amino acid. The combination of RNA editing and alternative splicing creates at least 25 proteins with different signaling properties (Fig. 5A). To determine changes in possible editing sites, we thus compared the A > G changes at the five editing sites. Because we sequenced more than 62 million reads per sample, this analysis was performed without any additional PCR amplification that was used in previous studies.

Similar to brain tissues,^41,42^ editing at the A site is most frequent, followed by editing at the B, D, C, and E and sites. Among the four different conditions, above and below the injury site, naïve and injured, there were no statistically relevant changes in overall editing (Fig. 5B).

When mRNA editing in individual fragments corresponding to individual RNA molecules was analyzed, simultaneous editing at the A, B, D sites followed by editing at AB and ABCD sites was most frequent. There was statistically significantly more editing at the CD sites in the injured caudal sections compared with all other conditions, but the overall editing at this site was very small (Fig. 5C,D).

When alternative splicing of exon Vb was analyzed, there was slightly more skipping of exon Vb in caudal injured segments, which, however, did not reach statistical significance (*p* < 0.3). There was also more skipping of exon Vb in caudal versus rostral segments, both for injured and naïve samples (Fig. 5E).

Thus, we found no evidence for widespread changes in the 5HT2C pre-mRNA processing in the rat model of S2 transection SCI.

## Discussion

### Detection of region-specific gene expression changes in a transection model

The changes in gene expression after SCI correlating with the development of chronic spasticity have been reported in various rodent models that assessed tissues either at or below the injury level.^5,11,43,44^ Using a complete upper sacral spinal cord transection model, we identified two major mechanisms altering gene expression under normal and injured conditions: transcription programs and the regulation of retained introns.

**FIG. 6.**
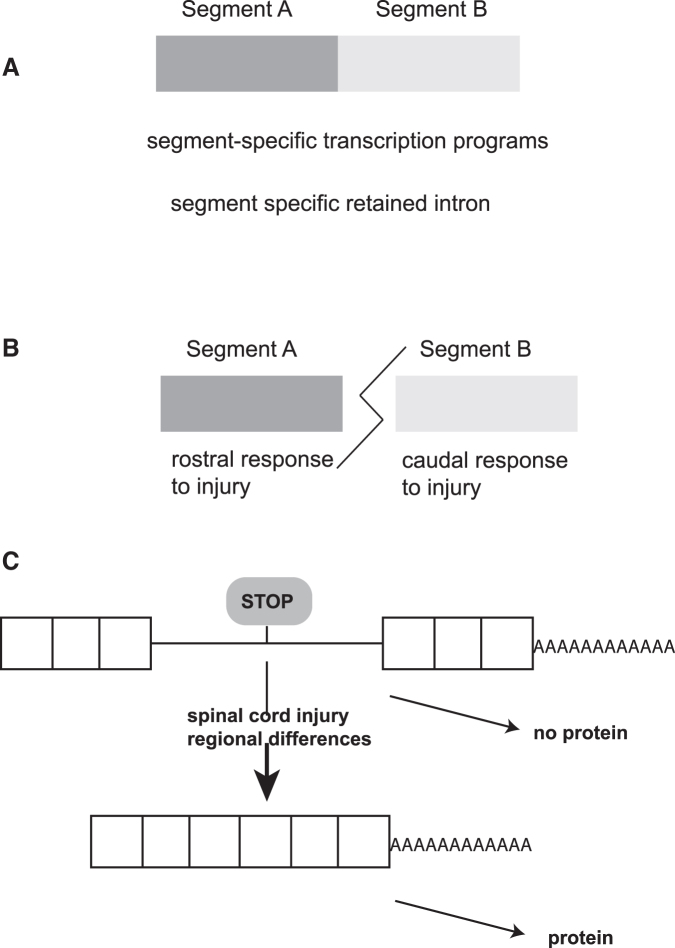
Model. (**A**) Under non-injured conditions, there are segment-specific transcription programs because of differential promoter usage in spinal cord segments. In addition, there are programs of differential retained intron regulation in genes distinct from the ones affected by promoter regulation. These introns reside in polyadenylated ribonucleic acid (RNA) that is likely retained in nuclei. (**B**) Injury elicits a rostral and caudal response, with the rostral response being stronger than the caudal response. (**C**) Spinal cord expressed numerous retained introns in polyadenylated RNA. Retained introns prevent protein formation because they likely reside in nuclei and, further, contain in frame stop codons. Their removal and subsequent messenger RNA (mRNA) translation of the mRNA depends on location and injury status.

Unexpectedly, the greatest differences were between rostral segments after injury, followed by differences between rostral and caudal segments under naïve conditions (Fig. 6A). We had predicted that there might be more changes caudal to the injury in parallel to remodeling of the lumbosacral neural circuity and fulminant development of chronic spasticity. Surprisingly, the segments rostral to the injury showed more gene expression changes compared with caudal areas (Fig. 6B). The function of these responses to injury remains to be determined. It is possible that the changes reflect an adaptation in gene expression to musculoskeletal changes after SCI.^45^

### Segment-specific differences in the lumbosacral spinal cord

In contrast to the brain composed of anatomically distinct areas, the spinal cord contains far more complex neural composition among the various segments. To this end, we found that the rostral versus caudal lumbosacral segments appeared to be distinct in their mRNA expression profiles, showing different transcriptional programs and novel polyadenylated RNAs with regulated retained introns. These segments also respond differently to injury at S2, with the rostral segments undergoing far greater changes in gene regulation.

While we anticipated seeing changes caudal to the injury in relation to spasticity, the data more likely reflect the heightened inflammatory, secondary degeneration, and wound healing responses in the intact cord rostral to the injury. Whether this is a global manifestation extending throughout the spinal levels of the neuroaxis or restricted to the lumbosacral spinal cord that we have investigated is yet to be determined.

### 5HT2C

It has been reported previously that S2 transection SCI leads to changes in 5HT2C editing, which could be an adaptation to the loss of serotonin delivery through the descending fibers leaving motor neurons in an unexcitable state.^4,5^ Employing the same S2 injury model, however, we did not find that these differences reach significance between comparable segments after injury.

A possible explanation for this difference could be different methods used. In the original report, cDNA was PCR amplified before assessment of the editing state, which we did not do in direct nanopore sequencing.

### Novel retained introns

In contrast to numerous stimulation paradigms in the central nervous system,^46,47^ we did not find changes in the alternative use of cassette exons. Surprisingly, we found that regulated intron retention is a possible new mode of gene regulation in the spinal cord. We identified more than 1,000 new retained introns. Similar introns have been identified previously in mouse cortex and mouse neuronal cultures.^25^ The introns are rapidly excised after a stimulus and their fully spliced mRNA transported into the cytosol and translated. This mechanism allows for rapidly produced mRNAs from large genes, which could take hours for complete processing. It is likely that a similar mechanism occurs in the spinal cord, allowing for rapid responses to neuronal stimulation in naïve spinal cord and possibly contributing to neuromuscular reflexes below the injury that are characteristic of spasms.

We observed strong differences in retained intron expression when comparing S1 and S2 fragments of naïve animals. With two exceptions, these differences are lost after injury, suggesting that the rapid response is region specific and affected by injury.

Because of the evolutionarily rapid changes in introns, we did not identify homologs at a larger scale. It is possible, however, that the regulatory principle is conserved. In this model, the retention of introns in nuclear polyadenylated RNA is conserved, because it allows rapid release of the mRNA, independent of a defined sequence (Fig. 6C).

The SCI and fulminant spasticity affect predominantly regulated retained intron retention in genes acting in RNA metabolism, mRNA splicing and transcription, suggesting that this is a newly discovered mode of transcript regulation in the spinal cord.

## Supplementary Material

Supplemental data

Supplemental data

Supplemental data

Supplemental data

Supplemental data

Supplemental data

Supplemental data

Supplemental data

## Abbreviations Used

Abca2: adenosine triphosphate binding cassette subfamily A member 2
AI: above injury
AN: above naive
ApoE: apolipoprotein E
ATP: adenosine triphosphate
BI: below injury
BN: below naive
COX: cytochrome c oxidase
DNA: deoxyribonucleic acid
EMG: electromyography
ETS: erythroblast transforming specific
Lgalo3: galectin 3
MBP: myelin basic protein
miRNA: micro ribonucleic acid
Mmp12: matrix metalloproteinase
mRNA: messenger ribonucleic acid
NADH: nicotinaminde adenine dinucleotide
PIR: percent intron retention
PCR: polymerase chain reaction
Reg3: regenerating islet-derived 3
RI: retained introns
RNA: ribonucleic acid
Scd2: stearoyl-CoA desaturase
SCI: spinal cord injury
SPARCL1: matrix associated protein
Ttr: transthyretin
