# Supplementary figures and images for: Rat Spinal Cord Injury Associated with Spasticity Leads to Widespread Changes in the Regulation of Retained Introns
Source: Neurotrauma Rep. 2022 Mar 4;3(1):105–21. doi: 10.1089/neur.2021.0042 (PMC8985541; doi:10.1089/neur.2021.0042)

A

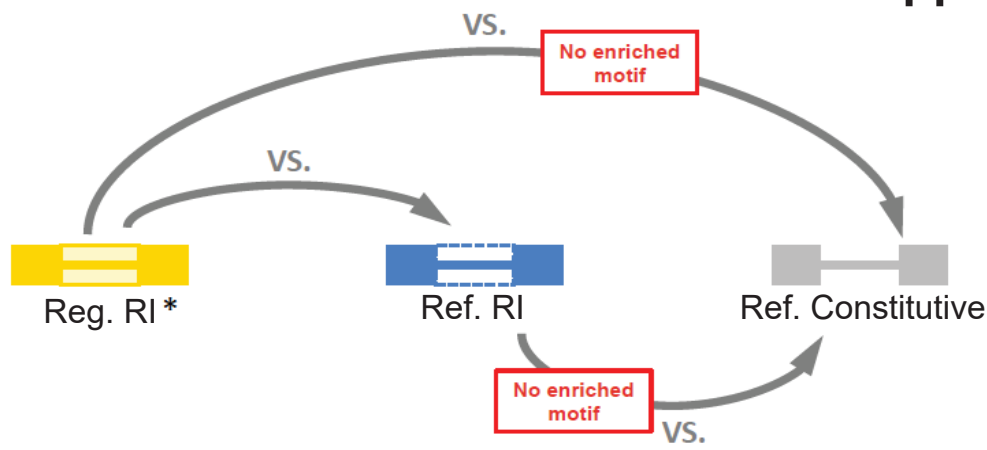

B

Proportion of sequences with motif

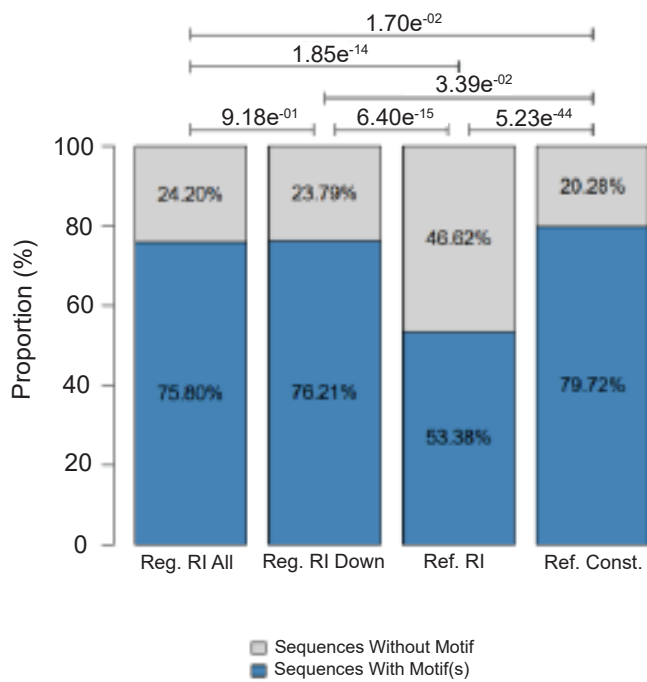

C

Number of motif by sequence

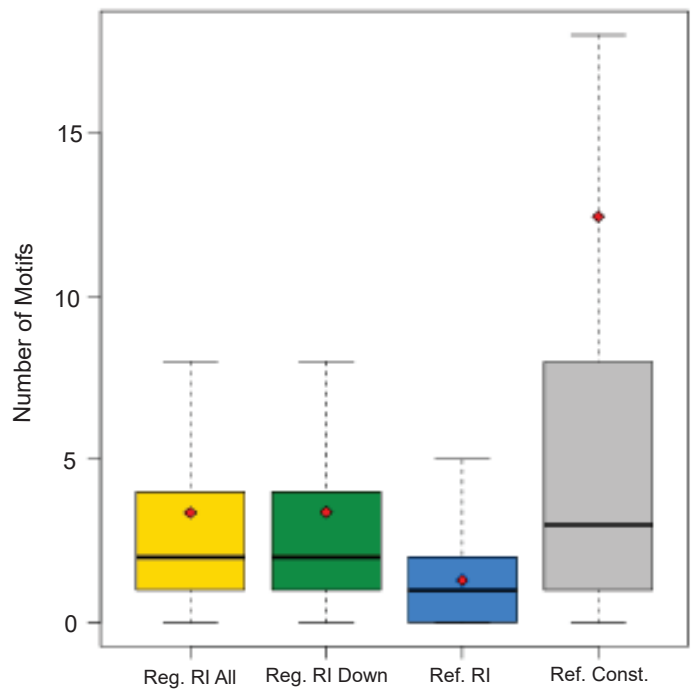

D

Distribution of Motif

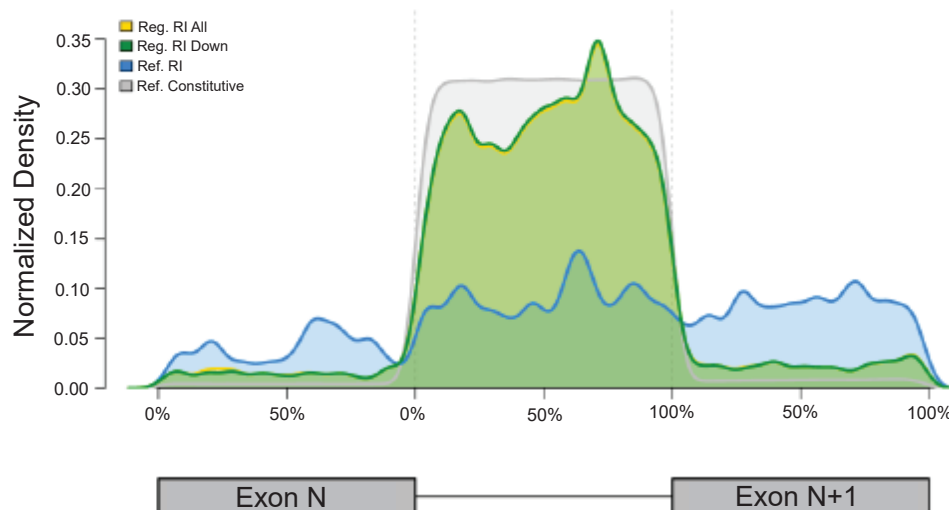

Supplement: Supplemental data [file Suppl_FigureS3.pdf]
